# Large scale matching of function to the genetic identity of retinal ganglion cells

**DOI:** 10.1038/s41598-017-15741-7

**Published:** 2017-11-13

**Authors:** Filippo Pisano, Erin Zampaglione, Niall McAlinden, Jennifer Roebber, Martin D. Dawson, Keith Mathieson, Alexander Sher

**Affiliations:** 1 Institute of Photonics, Dept. of Physics, University of Strathclyde, G1 1RD Glasgow, UK; 2 Molecular, Cell and Developmental Biology, University of California Santa Cruz, Santa Cruz, CA United States; 3 Santa Cruz Institute for Particle Physics, University of California Santa Cruz, Santa Cruz, CA United States

## Abstract

Understanding the role of neurons in encoding and transmitting information is a major goal in neuroscience. This requires insight on the data-rich neuronal spiking patterns combined, ideally, with morphology and genetic identity. Electrophysiologists have long experienced the trade-offs between anatomically-accurate single-cell recording techniques and high-density multi-cellular recording methods with poor anatomical correlations. In this study, we present a novel technique that combines large-scale micro-electrode array recordings with genetic identification and the anatomical location of the retinal ganglion cell soma. This was obtained through optogenetic stimulation and subsequent confocal imaging of genetically targeted retinal ganglion cell sub-populations in the mouse. With the many molecular options available for optogenetic gene expression, we view this method as a versatile tool for matching function to genetic classifications, which can be extended to include morphological information if the density of labelled cells is at the correct level.

## Introduction

The mammalian retina is a highly organized and approachable part of the central nervous system, which contains more than 60 distinct neuron types^1^ and provides some of the most elegant examples of how neural structure contributes to function^2^. The mouse retina, a system in which the investigation of neural circuits is empowered by a wide variety of genetic tools^3^, is an ideal platform to approach one of the fundamental goals of neuroscience; matching neuronal molecular composition and morphology with function.

Finding such a match is a demanding task that requires associating functional data with both high-resolution anatomical information and genetic identity. The latter often requires complex immunostaining and is subject to the availability of molecular markers.

Approaches such as single electrode^4,5^ and patch clamp^6–9^ have allowed significant advances in the comprehension of the retinal architecture, however, single cell recordings are limited in throughput. Recent advances in functional calcium imaging^10,11^ have overcome this problem, but lack the temporal resolution needed to characterize the precise temporal structure and interactions in spike trains from retinal neurons, parameters that are involved in the encoding of visual information^12^. On the other hand, microelectrode array (MEA) recording of retinal activity provides one of the best characterization methods of retinal response to visual stimuli at single cell resolution^13–16^. This area has seen significant technological development, particularly with the development of high-density, high-channel count CMOS MEAs^17,18^, yet studies do not yield direct information about the anatomical or genetic identity of the recorded Retinal Ganglion Cells (RGCs).

Recent work^19^ has reported anatomical identification of extracellularly recorded RGCs, where the spiking-induced electrical signature on an MEA (the Electrical Image, EI) was used to attribute electrophysiological signals to confocal images of anatomical somas. As the authors point out, this approach involves complex experimental procedures and success relies critically on the presence of a clear axonal image for each cell. This condition significantly limits the applicability of the match based solely on the EI and has motivated us to develop an innovative and accessible method to reliably match genetic identity to function in the RGC layer. Furthermore, we can identify the soma morphology/location and register this with confocal images that employ molecular staining protocols. We were unable to demonstrate a full morphological match that included the RGC dendritic structure, but conclude that this is possible with a sparser expression of labelled cells. To perform the functional match with genetic identity, we first targeted a particular sub-population of RGCs, using Cre-recombinase promoters^20,21^ to express a ChR2-tdTomato fusion protein. The functional response properties of the RGCs were measured by recording their *in-vitro* response to a visual stimulus using a 512-channel MEA^22,23^. We then pharmacologically blocked synaptic transmission in the retina and used a spatio-temporal optogenetic stimulation, performed with a high power μLED array^24^, to measure highly-localised, optogenetically-induced Spike Triggered Averages (OptoSTAs) of the cells expressing ChR2. Epifluorescent images of the retina on the MEA were taken to get soma locations of the ChR2-tdTomato-positive cells.

This approach gives the functional properties of the RGCs from their visual responses (recorded prior to the application of pharmacological blockers) and obtains the electrical image (EI) of the cells on the MEA. RGCs recorded pre and post application of the blockers are matched through their unique EIs. OptoSTAs of the ChR2-positive RGCs give an accurate spatial location of the individual cell body positions and allow the subsequent confocal imaging to link the partial anatomical information to function. Since ChR2 was transgenically expressed in subpopulations of RGCs, this also allowed us to attribute visual responses to a genetically distinct class of neurons.

The described method serves as a new, versatile, and approachable tool to establish a link between the molecular identities of the distinct retinal ganglion cell types and their function. With the appropriate density of labelled cells, we expect that standard confocal imaging techniques will also link the full morphological structure of the RGCs.

## Results

### Combining visual and optogenetic responses for the functional characterisation of genetically targeted RGCs

We have developed a system capable of performing spatio-temporal stimulation of visual pathways and direct optogenetic stimulation of retinal ganglion cells (RGCs). We demonstrate how the two stimulation paradigms can be combined to characterize the RGC functional response to normal visual stimulus and attribute this to an individual neuron through the direct optogenetic stimulation of the RGCs.

**Figure 1:**
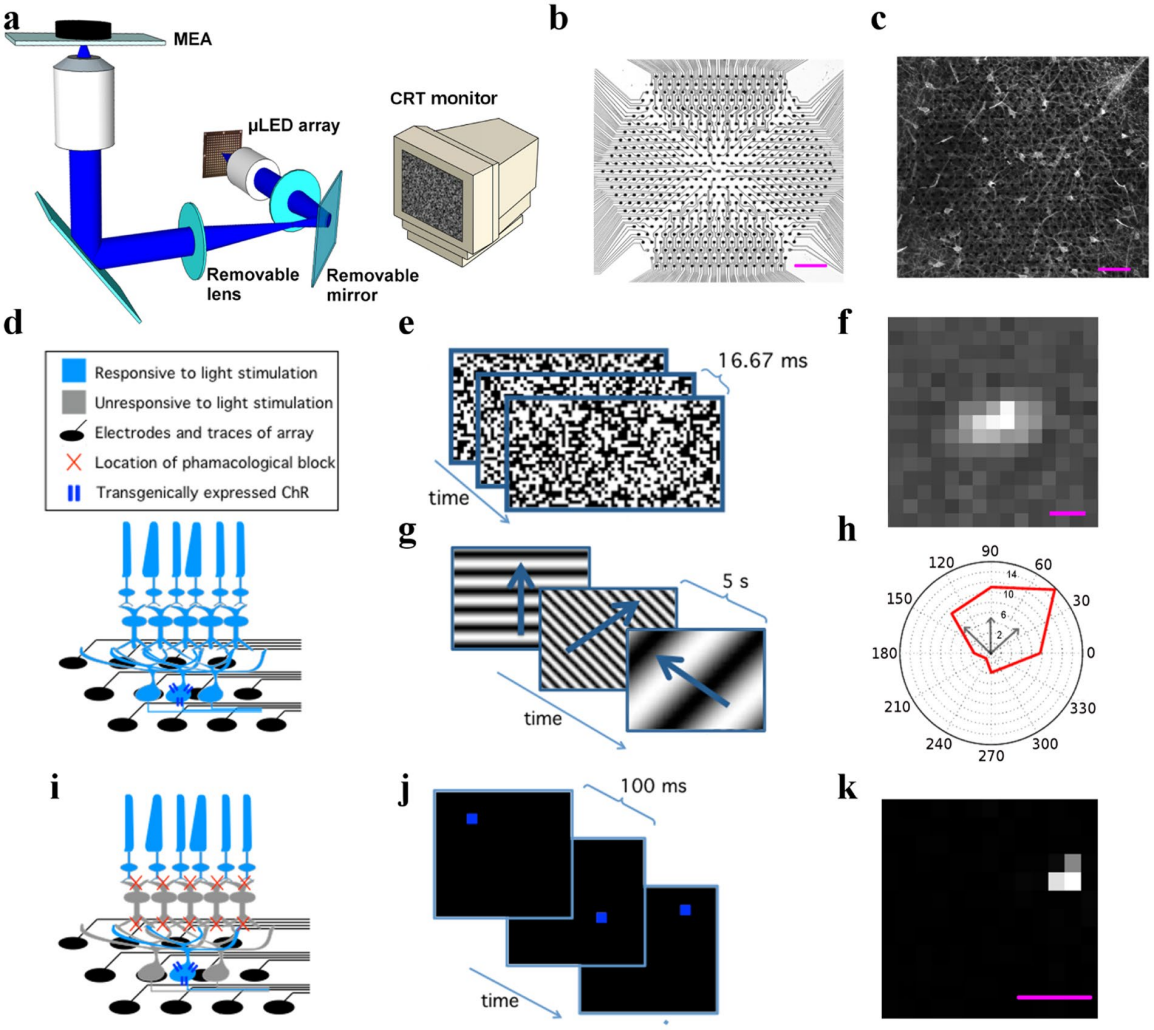
Both visual and optogenetic stimulation elicits spatio-temporally precise responses in RGCs on the MEA. (**a**) μLED projection system for optogenetic stimulation; a 16 × 16 array of square, 100 μm in size μLED pixels is imaged on the RGC layer with a 5x demagnification. The removable mirror and lens can be removed from the optical path, allowing visual stimulation of the retina by a CRT monitor (not to scale). (**b**) Detail of 30 μm pitch 512 MEA showing 5 μm diameter Pt electrodes and transparent ITO electrode tracks. (**c**) Grik4-Cre × Rosa26-lsl-ChR2-tdTomato retina mounted on the MEA. (**d**) Schematic of a retina placed over the MEA before pharmacological blocker application, when visual stimuli are used. (**e**) Example frames of the spatial-temporal white noise visual stimulus. (**f**) Example of visually induced ON spatial receptive field. (**g**) Stimulation of visual pathways with moving gratings of various spatial, temporal and directional parameters. (**h**) Example of a direction selective response, with the polar angle indicating the direction of visual stimuli and vector magnitudes representing spike rate (Hz) response to a given direction of motion. Three black arrows correspond to the directions of the gratings shown in panel (**g**). (**i**) Schematic of a retina placed over the MEA during pharmacological blocker application, when optogenetic stimuli are used. (**j**) Example frames of sparse white noise optogenetic stimulus. (**k**) Example of optogenetically induced spatial receptive field (OptoSTA). All scale bars are 100 μm. Schematic representations of retinal cells in (**d**,**i**) were modified from original drawings by Benjamin Stafford.

The MEA recording system was positioned on an inverted microscope equipped to accommodate both visual and optogenetic stimulation (Fig. 1a). We recorded from nasal-dorsal segments of mouse retina of different lines (see *Materials and Methods*) mounted RGC side down on the 512 electrode array (Fig. 1b,c).

Visual stimulation took place before the application of pharmacological blockers (Fig. 1d) and we used a spatio-temporal binary white-noise (Fig. 1e) to measure the spike triggered average (STA) responses of the RGCs^25^ (Fig. 1f). A moving gratings stimulus (Fig. 1g) allowed the measurement of RGC responses as a function of the stimulus motion direction (Fig. 1h).

Optogenetic stimulation took place during the application of pharmacological blockers (Fig. 1i), used to suppress the visual response mediated by photoreceptors.

We elicited action-potentials from ChR2-expressing cells (Grik4-Cre and CRH-ires-Cre retinas, see *Materials and Methods*) across the MEA with direct optogenetic stimulation of RGCs, obtained by focusing projected µLEDs pixels on to the retinal ganglion layer. The irradiance, in the plane of the RGCs, was optimized to a level where we consistently elicited a strong spiking response in the RGCs (~10 mW/mm^2^) through experiments on a Thy1-ChR2-YFP mouse strain. A sparse white noise movie was then presented to the retina with a single µLED pixel turned on per frame and the pixel position changing randomly in each frame (see *Materials and Methods*) (Fig. 1j).

Optically induced electrical artifacts, associated with the photoelectric effect, are common with optogenetic stimulation and complicate spike detection. This effect is particularly relevant to CMOS MEAs, where the control and amplifying circuitry can be affected by charge carrier generation from the illumination source^17^. However, we did not observe this effect in our system, due to the transparency of our electrode traces and shadowing of the electrode/electrolyte interface as we illuminated from the back of the array (Supplementary Fig. 1).

OptoSTAs were generated by reverse-correlating spike times with the spatio-temporal µLEDs stimulation sequence (Fig. 1k). In brief, we computed the average sequence of stimulating frames preceding a spike.

The OptoSTAs receptive fields were highly localized with the average area of (1.8 ± 0.1) ·10^3^ µm^2^ (5 experiments, 70 cells), an order of magnitude smaller than the average area calculated for Visual STAs, (3.3 ± 0.2) ·10^4^ µm^2^ (4 experiments, 857 cells). This highly localized response of the RGCs suggests the direct optogenetic activation of the cells.

Registering the OptoSTA position to the location of the imaged cells was accomplished by using the array of electrodes as a reference for both the cell images and the projected µLED position. As the next section will demonstrate, OptoSTAs were typically centred on the cell soma.

In summary, we have developed an experimental tool able to perform large-scale functional characterization of RGCs and subsequently generate highly spatially localised OptoSTAs within a genetically targeted sub-population of RGCs.

### Optogenetic identification of RGCs

The high level of ChR2 expression in the RGC somata should result in the spatial correlation between the OptoSTAs and the corresponding ChR2-positive cell bodies. In order to test if such correspondence could be established, we recorded from the retinas of two mouse lines where ChR2-tdTomato was expressed in distinct subpopulations of RGCs: Grik4-Cre × Rosa26-lsl-ChR2-tdTomato (4 retinas) and CRH-ires-Cre × Rosa26-lsl-ChR2-tdTomato (1 retina).

**Figure 2:**
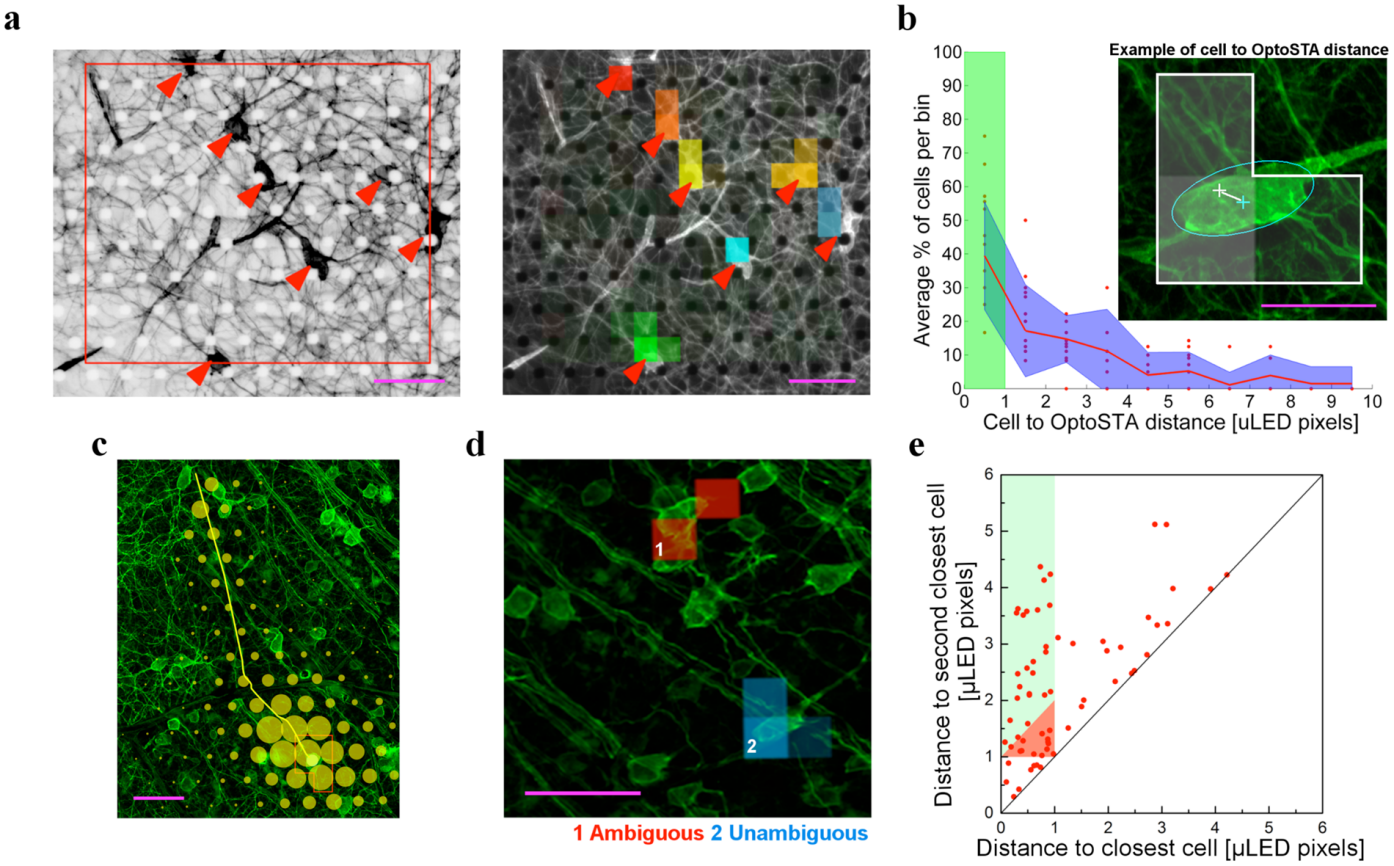
Optogenetically-induced spatial receptive fields (OptoSTAs) colocalize with the RGC cell body locations. (**a**) *Left*, Fluorescence image of RGCs from Grik4-Cre × Rosa26-lsl-ChR2-tdTomato retina (greyscale inverted); stimulated area is outlined in red (~280 × 300 µm^2^), cell bodies are indicated by red arrowheads. *Right*, Coloured OptoSTAs are overlaid on the photograph shown in the left panel: location of each OptoSTA is closely correlated to the location of the cell bodies. Intensity of OptoSTA pixels is proportional to the correlation with spiking activity. Scale bars are 60 μm. (**b**) Histogram of the distances from the OptoSTA location of the recorded cell to the cell body of the nearest fluorescent cell. Cells within 1 μLED pixel from an OptoSTA pass the acceptance cut (green shaded area); averages from each of the recordings are represented as red dots while standard deviation across datasets (11 across 5 experiments) is shaded in blue. *Inbox*, Example of a cell to OptoSTA distance: in white is the OptoSTA outline with the centre of gravity shown as a white cross; in cyan are the ellipse encompassing the cell and its centroid; scale bar is 25 μm. (**c**) An example of an electrical image matching the associated cell’s axon trajectory. Yellow circles represent the electrodes location; the circles diameter is proportional to the recorded voltage deflection. The somatic amplitudes are saturated due to the scaling (x4) of all amplitudes to make the axonal signals more visible. Scale bar is 60 μm. (**d**) Optogenetic identification: example of a discarded match, in red, and a successful one, in blue; scale bar is 60 μm. (**e**) Scatter plot (5 experiments) of OptoSTAs’ centres of gravity distance from the closest (x axis) and second-closest (y axis) ChR2-expressing cells. Distances are measured in μLED pixel size. Data points falling in the green-coloured area (x < 1, y > x + 1) qualify as an unambiguous optogenetic match. Scale bar is 60 μm.

We found that spatial positions of OptoSTAs are highly correlated with the spatial locations of ChR2-expressing RGC somas and so allow electrophysiological data, in the form of spike trains, to be attributed to the individual labeled RGCs (Fig. 2a). To quantify the correlation between OptoSTAs and RGCs’ soma position we calculated the distribution of the distances from the OptoSTA location for each recorded cell to the cell body of the nearest fluorescent cell (Fig. 2b, inset). OptoSTA locations were defined by computing a centre of mass of the OptoSTA (*see Materials and Methods*); cell soma position was estimated by the centroid of a manually fitted ellipse encircling the fluorescent image of the cell body. Any cell soma located outside the area covered by the microLED stimulation was dismissed from the analysis *a priori*.

In 5 retinal preparations and a total of 11 optogenetic stimulation runs (*see Materials and Methods*), we illuminated ~120 ChR2-expressing cells and identified spike trains from 70 RGCs that also had good OptoSTAs (contained within 4 or less µLED pixels - *see Materials and Methods*). As shown in Fig. 2b, nearly half of the ChR2-positive cells (39 ± 5%, standard error of the mean) fall within 1 μLED-pixel distance from an OptoSTA (green area in Fig. 2b). In order to verify the position match between the labelled and recorded cells we identified fluorescent cells for which we could trace the axon. The anatomical axon trajectory was overlaid with the electrical counterpart from the recorded cell, and the close alignment provides further confirmation that the OptoSTA receptive field did indeed correspond to the soma location. An example of a cell with an identified axon is shown in Fig. 2c. Only a few such cells (5–10) were found where axons were clearly attributable to a soma in the fluorescence images. This scarcity of traceable axons is typical and gives further motivation to our OptoSTA technique, which does not rely on axon tracing. It is important to note that the neurons we considered for the analysis were driven by somatic stimulation only. None of the RGCs with significant OptoSTAs had the signal backpropagation from the axon to the cell body, as assessed by manual inspection of the EIs^26^. Thus we saw no clear evidence for axonal stimulation throughout the datasets.

While our approach ensures that the cell with an OptoSTA can only be a ChR2-tdTomato-positive cell, the finite projected μLED pixel size (23 μm side) leads to ambiguity in the match between OptoSTAs and fluorescent cell bodies if two or more labelled cells are located close to each other: examples are displayed in Fig. 2d.

This ambiguity can be removed by manually monitoring OptoSTA-soma distances to check if they generate a satisfying match with the ChR2-expressing cell. However, applying such an approach over tens or hundreds of cells is time consuming and might suffer from subjective bias. In order to objectively identify unambiguous matches between OptoSTAs and ChR2-expressing cells, we considered the distances between each OptoSTA and the neighbouring cells. For each OptoSTA, we calculated the distances to the nearest and second-nearest labelled cell body and then introduced an additional cut (Fig. 2e), where we let D_1_ be the distance to the closest cell, D_2_ be the distance to the second closest cell and L be the lateral dimension of a projected µLED pixel (23 µm). We considered any OptoSTAs-cell pair with 0 < D_1_ < L and D_2_ > D_1_ + L to be an “unambiguous match”. This acceptance area is coloured green in Fig. 2e. No match could be established for pairs lying in the region next to the origin, 0 < D_1_ < L and 0 < D_2_ < L, which we defined as a rejection area.

A transition region, 0 < D_1_ < L and L < D_2_ < D_1_ + L, exists between the areas of acceptance and rejection where ambiguous and unambiguous matches are contiguous, as we could observe with a case-by-case analysis. In order to avoid any weak match, we opted for a conservative approach and discarded all pairs lying in this area.

Using the described criteria and starting from 70 physiologically recorded RGCs with good OptoSTAs (5 experiments), we could unambiguously link 30 of them to Cre-targeted anatomical RGCs. It should be noted that a higher density of ChR2-expressing neurons resulted in a higher rejection rate (Fig. 2e). Comparing two experiments performed on different genetic lines, we found that stimulation runs over 5 areas of a Grik4-Cre retina, targeted an average of 14 cells per stimulation area (70 cells in total) and resulted in 12 rejections; at the same time, stimulation runs over 3 areas in a CRH-ires-Cre retina, targeted an average of 9 cells per stimulation area (27 in total) and resulted in 3 rejections. For the cells with good OptoSTAs, we compared visually and optogenetically induced electrical images. As reported in the next section, this technique allows the establishment of a link between genetic identity and visual function. In conclusion, we have proven that a sparse-white-noise optogenetic stimulation approach allows linking electrophysiologically identified cells with their anatomical soma.

### Matching function with genetic identity

In order to find the visual response properties of the ChR2-positive cells identified in the previous section we used electrical images (EIs). As mentioned above, the EI of a cell is the unique electrical footprint of the RGC’s spiking activity recorded on the MEA and is independent of the method of stimulation used to elicit the RGC’s spiking (*see Materials and Methods*). Therefore, matching optogenetically-elicited and visually-elicited EIs in the same preparation links genetic identity, soma location and the visual response properties of the RGCs. In order to achieve this, we cross-correlated the optogenetically-elicited EIs with their visual counterparts. In particular, we compared the optogenetically-elicited EIs of the RGCs that had good OptoSTAs, with the visually-elicited EIs of the RGCs, recorded prior to the application of neurotransmitter blockers. All possible pairs of the EIs were considered and for each pair the degree of similarity was estimated by the normalized inner product (IP) between the two EIs (*see Materials and Methods*). Similar EIs with IP close to 1 corresponded to the same RGC recorded across the two datasets (optogenetically-induced and visually-induced). An IP value closer to 0 pointed to a pair of dissimilar EIs corresponding to two different RGCs.

We used the IP values for the best and the next best matches as the criteria for acceptance. More precisely, if IP_1,2_ denotes the inner product between the two EIs and IP_1,n_, IP_2,m,_ the IP values between each of the paired EIs and their respective second closest EI, then the original pair of EIs was accepted as a match if IP_1,2_ > 0.95 and IP_1,n_ < IP_1,2_ −0.05, IP_2,m_ < IP_1,2_ −0.05. An example of the distribution of correlations among the tested pairs is shown in Supplementary Fig. 2.

**Figure 3:**
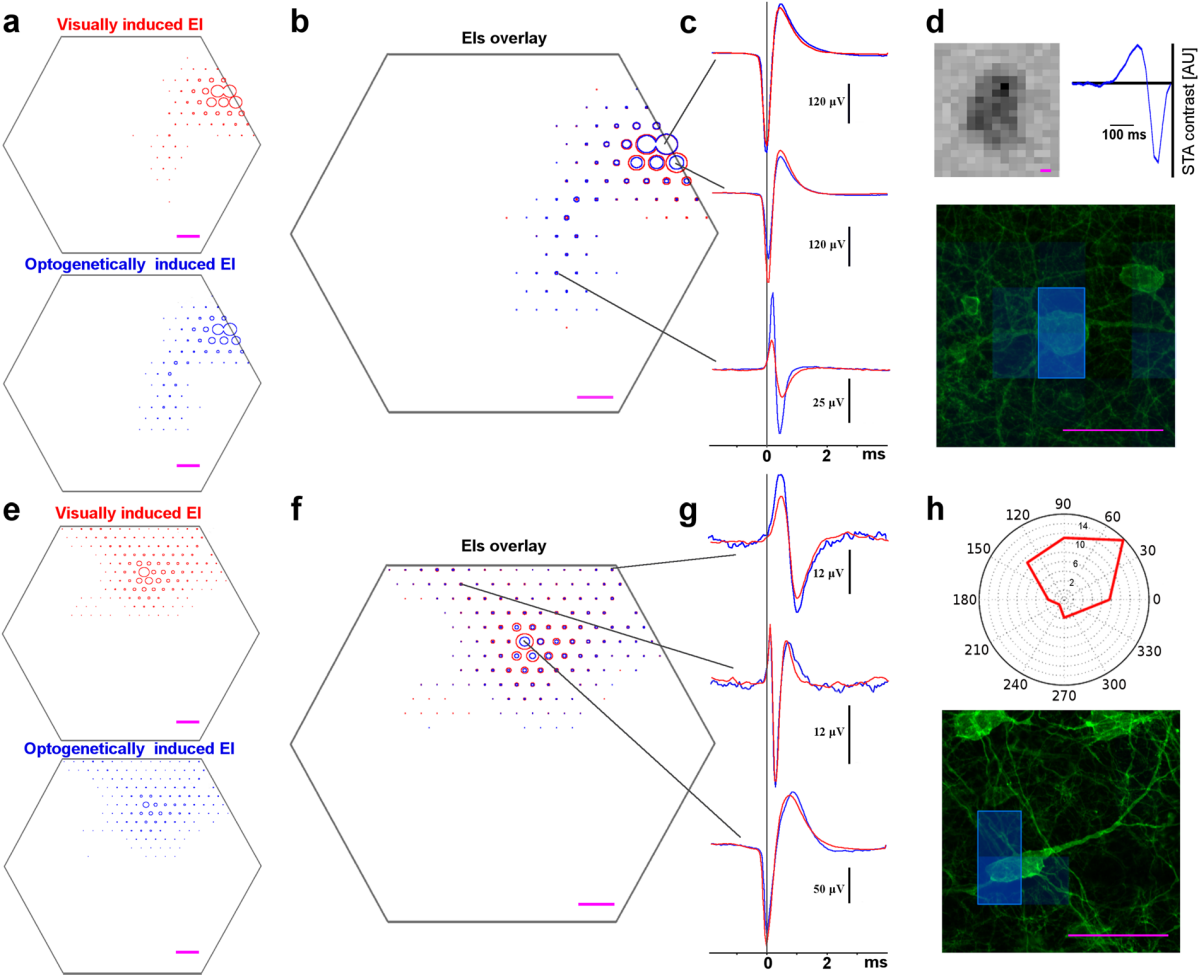
Matching confocal images of RGCs to their function. (**a**) Visually induced EI, *top*, and optogenetically induced EI, *bottom*, for a CRH-ires-Cre-expressing RGC; the MEA is outlined in grey. (**b**) Overlay of EIs: optogenetically induced EI in blue and visually induced EI in red. (**c**) Comparison of spike waveforms recorded across the MEA: optogenetically induced trace in blue and visually induced trace in red. (**d**) *Top:* Visual response of the RGC from (**a**) and (**b**): STA receptive field (the STA frame with the largest contrast at 48 ms before the spike) and the timecourse (STA amplitude within the receptive field as a function of time). *Bottom*: confocal image with outlined OptoSTA of the same cell. (**e**) Visually induced EI, *top*, and optogenetically induced EI, *bottom*, for a Grik4-Cre-expressing RGC; the MEA is outlined in grey. (**f**) Overlay of electrical images: optogenetically induced EI in blue and visually induced EI in red. (**g**) Comparison of spike waveforms recorded across the MEA: optogenetically induced trace in blue and visually induced trace in red. (**h**) Visual Direction Selective response of the RGC from (**e**) and (**f**), *top*, and confocal image with outlined OptoSTA of the same cell, *bottom*. All scale bars are 5 μm.

Examples of successful EI correlation and consequent matching of genetic identity and function are shown in Fig. 3. We demonstrate the validity of the EI matching process by comparing the full voltage waveforms on select electrodes (Fig. 3c–g). We found that it is enough to rely only on correlating the maximum voltage deflection value on each electrode to perform an unambiguous match between optogenetically and visually induced EIs. The exact EI waveform on each electrode did not improve the match. The CRH-ires-Cre-positive RGC in Fig. 3 had OFF-center response properties based on the white noise stimulation (Fig. 3d) and the Grik4-Cre example RGC had a direction-selective response to motion (Fig. 3h).

After the recordings, we performed a standard staining protocol to amplify the fluorescent signal (*see Materials and Methods*) and imaged the recorded retinas on a confocal microscope. Examples of the spatial correlation between the somas of the labelled RGCs and the OptoSTAs of the matched RGCs recorded on the MEA are shown in Fig. 3d,h. The density of the labelled cells in both CRH-ires-Cre (Fig. 3d) and Grik4-Cre (Fig. 3h) retinas led to the processes (dendrites and axons) of different cells overlapping to the degree that did not allow reconstruction of the dendritic trees of the individual RGCs beyond the proximal dendrites.

**Figure 4:**
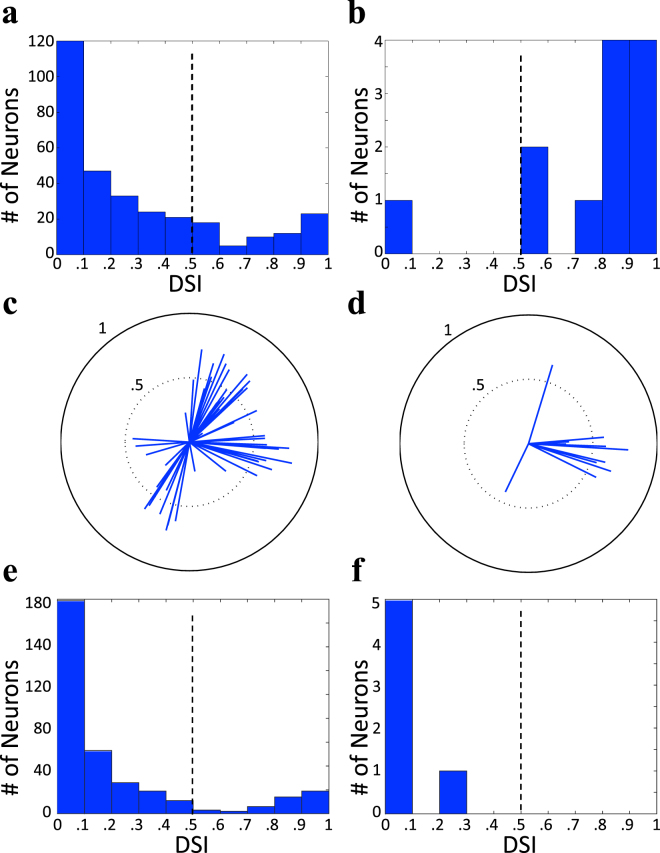
Direction of motion selectivity differs in optogenetically responsive cells in the Grik4-Cre and CRH-ires-Cre-expressing RGCs. (**a**) Distribution of DSIs for all visually responsive cells recorded in a single Grik4-Cre × Rosa26-lsl-ChR2-tdTomato retina (314 RGCs). (**b**) Distribution of DSIs for optogenetically responsive cells in the same retina (12 optogenetically active RGCs). (**c**) DS vectors for all visually active cells with DSI > 0.5 in the Grik4-Cre × Rosa26-lsl-ChR2-tdTomato retina (64 DS RGCs). (**d**) DS vectors for all optogenetically active cells with DSI > 0.5 in the same retina (11 DS RGCs). Each DS vector is the vector sum of a single RGC’s responses to different directions of the stimulus motion, normalized to one. (**e**) Distribution of DSIs for all visually responsive cells recorded in a single CRH-ires-Cre × Rosa26-lsl-ChR2-tdTomato retina (332 RGCs). (**f**) Distribution of DSIs for optogenetically responsive cells in the same CRH-ires-Cre × Rosa26-lsl-ChR2-tdTomato retina (6 optogenetically active RGCs).

In order to measure the functional properties of the Grik4-Cre-positive RGCs we used the single best Grik4-Cre recording in which we had 70 fluorescent cells within the field of view of the μLED array, and 40 OptoSTAs to analyse DS response following a previously described approach^18^. For 13 of the 40 cells we found EI-correlates in the data run where retinal responses to moving gratings were recorded. Almost all of these cells (12/13) had direction selective (DS) responses (defined as having a direction selective index, DSI > 0.5 - *see Materials & Methods*). In contrast to this, only 20% (64/314) of all visually responsive RGCs (*see Materials and Methods*) identified in the same recording had direction preference (Fig. 4a,b).

Furthermore, we found that while visual stimulation (Fig. 4c) elicited direction selective responses in all four cardinal directions, optogenetic stimulation (Fig. 4d) generated responses mainly from a single direction. The null hypothesis of no preferred direction for Grik4-Cre-positive cells has been rejected with a Student’s t-test (t > 2.13, significance level = 0.05, 4 dof, *see Materials and Methods*). We did not track the orientation of the retina during the recording and therefore could not determine which single cardinal direction of motion was preferred by the identified RGCs.

Note that these results were obtained without imposing the strict cuts on the spatial match between OptoSTAs and the labeled RGC soma locations. These cuts would decrease the initial pool of 40 RGCs with good OptoSTAs to 14. Thus, the requirement of spatially identifying the exact labelled cell reduces the efficiency of the described approach. However, if the aim is simply to attribute function to a genetic sub-class of neurons, then the previous set of statistics stand.

To demonstrate this technique in a different genetic line (CRH-ires-Cre), we illuminated a region covering 27 fluorescence cells and obtained 11 OptoSTAs. From these OptoSTAs we produced 11 optogenetically-induced EIs, which matched to 7 visual EIs in the white noise dataset (3 OFF cells and 4 ON cells). 6 of these cells were also matched with visual EIs from the drifting gratings dataset. None of these cells showed direction selective responses (Fig. 4e,f).

## Discussion

We have described a method to associate functional data from large-scale extracellular electrophysiological recordings with genetically labelled RGCs in mice and shown how spatio-temporal optogenetic stimulation can be employed to generate highly-localised receptive fields that unambiguously identify individual RGC somas. We demonstrated that this experimental protocol could be combined with a functional characterization procedure based on the presentation of spatio-temporal visual stimuli. Furthermore, visual characterisation and optogenetic identification do converge, in a single analytical framework, allowing a correlation of function and genetic identity in the recorded neural population. Partial morphological information was also obtained through confocal images of the RGC somas. We expect that a reduced density of labelled cells will allow standard image reconstruction techniques to be employed, potentially adding dendritic tree morphology to this data. Immunostaining for additional molecules, for example transcription factors known to be expressed in specific RGC types^27^, can be performed on the retina after the electrophysiological recording to enrich the information about the molecular identity of the identified cells. Furthermore, it may be possible to inject some or all of the matched cells with fluorescent dye to assist with tracking the processes, even in densely labelled retinas. This would require identification and injection of the matched cells before any retinal deterioration begins. At the moment, the spike sorting and STA calculation is performed almost in real time. Therefore, streamlining the matching procedure, which involves uncomplicated image processing, should make it possible to identify the matched cells in a suitable timeframe.

The findings of our study are consistent with previous characterizations of the transgenic mouse lines. We saw a fraction of the Grik4-Cre-expressing cells show a selective response to motion in a single direction agreeing with previous studies showing a fraction of these cells to be selective for anterior motion^28^. Conversely, none of the CRH-ires-Cre-expressing RGCs had a DS response, which is consistent with the finding that CRH-ires-Cre generally expresses in displaced amacrine cells and mono-stratified RGCs^29^.

OptoSTAs of RGC containing channelrhodopsin have been generated by recording responses of RGCs to a random sequence of single μLED pixels. These receptive fields corresponded to the spatial locations of the ChR2-expressing cells visualized by fluorescent microscopy. This correspondence allowed us to connect the individual electrophysiologically recorded RGCs to the single cells within a genetically targeted sub-population. The described technique is accessible and versatile being based on a simple optical architecture. However, as shown in Fig. 2b not all the ChR2-expressing cells have an associated OptoSTA. This can, perhaps, be attributed to three factors. Firstly, the limited photosensitivity of ChR2-expressing neurons in the Ai27 line^30^ might have affected the stimulation efficiency. We therefore think our method will give better results if Ai32 ChR2 reporter mice are used. Secondly, it is not unreasonable to assume that a small portion of the ChR2-expressing cells may have gone undetected despite the high-density MEA. Thirdly, the well-recognised limitations of current spike-sorting algorithms^31^ removes a number of cells, due to classification effects such as cross-contamination between single units. For example, ~40 neurons with good OptoSTAs were rejected out of the ~130 identified across 5 experiments due to this type of contamination. Advances in spike sorting will undoubtedly improve the efficiency of identifying ChR2-positive cells.

Matching of function to the genetic identity has been achieved by correlating the unique and stimulus-independent electrical signatures of spiking activity induced by the RGCs on the MEA. As previously observed^32^, spike amplitude tends to diminish during an optogenetically excited burst, potentially leading to smaller average amplitudes in the electrical image. The changes in the RGC physiology over the course of the experiment can also lead to changes in the signal amplitudes. We therefore chose a loosely scale-dependent parameter (inner product) to correlate EIs between the visual and the optogenetic stimulus recordings. However, while the amplitude of optogenetically and visually induced spikes might differ, the high temporal correlation of spike waveforms (Fig. 3) gives further reassurance of exact matching. As reported in the result section, we applied strict significance cuts in matching EIs in order to ensure a unique and unambiguous match. Relaxing these statistical constraints as well as using alternative methods for finding spikes associated with unique neurons across varying stimulation conditions could be investigated in order to optimize the method.

The anatomical identification of extracellularly recorded RGCs in primate has been recently reported in a study by Li *et al*.^19^ who demonstrated a different approach that relied on the electrical image alone. The method described in our study has the following advantage; if the OptoSTA receptive field overlaps with the single labelled cell soma and there are no additional labelled cells nearby, the match between the electrophysiologically identified cell and the anatomical cell soma is unambiguous, because all nearby RGCs lack ChR2 and would not respond to the optogenetic stimulation. Conversely, there is no such certainty when matching electrophysiology to labelled RGCs using only electrical images. Instead, one has to rely on the precise match between the labelled cell’s axon and the EI axonal trajectory and on the functional and morphological properties of the matched cells. Unfortunately, axonal trajectories of the individual labelled cells are often impossible to track even with the help of confocal microscopy. The functional and morphological properties of the cells are useful only if the match between these two characteristics has been established *a priori*. Our method avoids these limitations. Nonetheless, the two experimental paradigms are not mutually exclusive and can be combined. For example, even if a few ChR2-expressing cells are located next to each other, but their individual axons can be traced, the correlation of the axons with the axonal trajectory of the EI of the candidate optogenetically activated RGC can be used to resolve the ambiguity.

The described technique is a promising tool for a comprehensive functional characterization of already described and newly discovered anatomical and molecular RGC types. In fact, the method can be employed across a wide range of expression patterns of ChR2 in RGCs. For example, the reporter we utilised (a Cre-dependent ChR2-tdTomato fusion protein at the Rosa26 locus) could be combined with virally expressed Cre-recombinase for random and sparse labelling^21^ or with the recently described Cre-DOG system that allows Cre to be expressed in transgenic GFP lines^33^. A diverse and valuable approach would focus on a sparse labelling technique using a viral reporter^29^. The density of expression of ChR2 is a key parameter that must be considered while designing an experiment that applies the reported technique to the characterization of a particular genetic model. The closest distance at which two ChR2-expressing cells can be distinguished by their OptoSTAs is dictated by the size of the projected pixels, as shown in Fig. 2d,e. This resolution distance can be readily improved by using smaller light sources or by optically reducing the size of the projected μLEDs. The single μLED size used in this study was chosen as a trade-off between resolution and the size of the stimulated area. However, on-going developments of the μLED arrays with larger number of pixels promises larger area stimulation at higher resolution that will result in higher efficiency of unambiguous match between anatomy and function. Finally, the generation of cellular specific OptoSTAs might serve as a valuable tool to investigate connections between anatomy and function in neural circuits other than the retina.

## Materials and Methods

### Mice

Animals were cared for and used in accordance with guidelines of the U.S. Public Health Service Policy on Humane Care and Use of Laboratory Animals and the NIH Guide for the Care and Use of Laboratory Animals. The University of California Santa Cruz Institutional Animal Care and Use Committee approved all experimental procedures used for this research. All mice were purchased from Jackson Laboratories and backcrossed to C57Bl/6 lines in the author’s laboratory. Genotyping was done using DNA from a tail clip. Initial testing was done on Thy1-ChR2-YFP strains (JAX 007615). Animals were identified as positive for the transgene with primers against GFP (5′-CCTACGGCGTGCAGTGCTTCAGC-3′ and 5′-CGGCGAGCTGCACGCTGCGTCCTC-3′). Two Cre lines were used: CRH-ires-Cre (JAX 012704) and Grik4-Cre (JAX 006474).

Animals were identified as positive for their transgenes with primers against CRH-ires-Cre (5′- CAATGTATCTTATCATGTCTGGATCC-3′ and 5′-CTTACACATTTCGTCCTAGCC-3′) or a general Cre primer set (5′-ACCAGAGACGGAAATCCATCG-3′ and 5′-TGCCACGACCAAGTGACAGCAATG-3′). Cre lines were crossed to a Rosa26-lsl-ChR2-tdTomato (JAX 012567) strain. Mice were identified as positive for this transgene with primers against tdTomato (5′-CTGTTCCTGTACGGCATGG-3′ and 5′-GGCATTAAAGCAGCGTATCC-3′).

### Electrophysiology Recording

Eyes were enucleated from terminally anesthetized mice (intraperitoneal injection of 16 mg/mL ketamine and 4 mg/mL xylazine in PBS) after 20 minutes of dark adaptation. The eye dissection was performed in oxygenated Ames’ solution (Sigma). A circular cut around the eye at the border between sclera and cornea was done with small spring scissors. After this, the anterior portion of the eye and the vitreous were removed. A portion of the retina and sclera from dorsal or nasal part of the eye was cut off the eyecup with a scalpel. From that piece, the retina was separated by carefully peeling it from the sclera. The resulting retinal piece was further trimmed down to 1 to 3 mm^2^ size. It was then placed onto the microelectrode array chamber filled with Ames’ solution. After positioning the retina over the microelectrode array with the retinal ganglion cell (RGC) side down, it was pressed down against the electrodes with a dialysis membrane (Fisher Scientific cat. No. 08-670-3BB) strung on a custom-made plug. Eye removal and retinal dissection were performed under dim red light. While recording, retinas were perfused with Ames’ solution (34 ± 2 °C) bubbled with 95% O_2_, 5% CO_2_, pH 7.4. An MEA consisting of 519 electrodes with 30 μm spacing was used for the recordings^22^. The electrodes, arranged in an isosceles triangular lattice, covered a hexagonal region of 450 μm on a side; 7 out of 519 electrodes were disconnected. Voltage signals were digitized at 20 kHz per channel with custom designed integrated circuits and stored for offline data analysis^23^.

### Visual stimulation

Responses to visual stimuli (spatio-temporal white noise and moving gratings) were measured using low scotopic light levels that did not activate ChR2. The movies were produced using a custom LISP code and displayed on a CRT monitor with refresh rate of 120 Hz.

The stimuli used were 1) a binary spatio-temporal white noise with a pixel size of 45 × 45 um^2^ and a frame rate of 60 Hz, and 2) a series of moving sinusoidal gratings in eight equally spaced directions with three spatial periods (576 μm, 1152 μm, 2304 μm) and three temporal periods (0.53 sec, 1.07 sec, 2.13 sec) for a total of 72 unique parameter combinations. Epifluorescence images of the recorded retinal sections (Fig. 1c) were acquired at different magnifications (5X, 10X, 20X).

### Optogenetic Stimulation

To unmask the direct activation of ChR2-positive RGCs, we inhibited the signal transmission from photoreceptors to RGCs with a mixture of pharmacological blockers (DNQX at 150 µM, DL-AP7 at 200 µM, L-AP4 at 50 µM, Kynurenic acid at 1 mM, Picrotoxin at 50 µM and Strychnine at 50 µM).

Sparse white noise optogenetic stimulation of ChR2-positive RGCs was performed by projecting a 16 × 16 high-intensity (~10 mW/mm^2^), blue (450 nm) µLED array^24^ onto the RGC layer. Square µLED pixels were optically reduced to ~23 × 23 μm^2^ in size and focused on the RGC layer for 50 ms-long pulses repeated every 100 ms. Frames of the sparse-white-noise LED stimulation movie consisted of a single pixel turned on at a random position every frame. The size of the projected µLED array was ~280 × 300 μm^2^, only a fraction of the 0.4 mm^2^ active area of the MEA. Thus, in order to maximize the yield from a single retinal recording, multiple optogenetic stimulation runs were performed at different locations of the recorded retinal sections by moving the MEA with respect to the stimulating objective using a manually controlled microscope stage. LED position, with respect to the electrodes, was recorded by imaging locations of the projected LED pixels on the electrode array before and after each stimulation.

### Electrophysiology Data Analysis

Recordings were analysed in order to segregate spikes of different RGCs as previously described^15,23^. Briefly, the shapes of the voltage waveforms of the RGC action potentials (spikes) recorded on the multiple electrodes of the dense MEA were used to identify spike trains from individual RGCs. Refractory period violations were used to eliminate RGCs with contaminated spike trains. In particular, the cells with more than 10% contaminating spikes were excluded with the contamination estimated from the number of the refractory period violations and assuming that the contamination is stochastic. Electrical images (EIs) for the recorded cells were calculated by averaging the voltage waveforms recorded on all the electrodes when the RGC produced a spike. For each spike, a 5 ms time-window centred on the peak negative deflection of the voltage waveform was considered. A 2D plot of the maximal absolute voltage deflection at each electrode site was used as a spatial representation of the EI (Fig. 3). Spatial receptive fields and response dynamics of the identified RGCs were measured by calculating their spike triggered average (STA) response to the binary white noise and sparse white noise stimuli for the visual and optogenetic stimulation respectively. Direction selectivity in response to moving gratings was calculated as previously described^34^. Briefly, direction selectivity was characterized by computing the Direction Selectivity Index (DSI) for every spatial/temporal combination of moving gratings stimuli. The DSI was computed as

1 $${\rm{DSI}}=\frac{{\rm{pref}}-{\rm{null}}}{\mathrm{pref}\,+{\rm{null}}}$$

where pref corresponds to the average spike rate in the cell’s preferred direction, and null corresponds to the average spike rate in the direction 180° opposite to the preferred direction. If one of the nine spatial/temporal combinations had a DSI > 0.5, the cell was considered to be direction selective.

Optogenetic spike-triggered average responses (OptoSTAs) to the sparse white noise stimulus were used to locate the soma positions of ChR2-expressing cells. OptoSTAs were calculated by reverse correlating the spike times recorded from the RGCs with the spatio-temporal LED stimulation. In order to determine which pixels elicited a significant optogenetic response, we set a threshold of 5 times the root mean square (RMS) intensity value calculated from the pixels to pixel variation across the OptoSTA frame. The same significance cut-off was used to calculate the number of significant pixels within the OptoSTAs. In each case, the OptoSTA center of mass was calculated using the intensity values of all the significant pixels in the OptoSTA frame preceding the spike.

The RGCs separately identified in the visual and optogenetic stimulation runs were matched by comparing their EIs. In particular, for all possible pairs of EIs, the normalized inner product was calculated as:

2 $$IP(E{I}_{1},E{I}_{2})=\frac{{\sum }_{e}{O}_{e}\cdot {V}_{e}}{\sqrt{{\sum }_{e}{O}_{e}^{2}+{\sum }_{e}{V}_{e}^{2}}},$$

where O_e_ and V_e_ are optogenetically and visually evoked average spike amplitudes, recorded on each electrode *e* with SNR > 10, normalized to the maximum amplitude detected in the EI. The normalization was done to make the comparison depend more on the shape of the averaged spike waveform and not on its amplitude.

A two-step cut was applied to identify matching EIs. First, an EI pair had to have a normalised inner product greater than 0.95 to be accepted as a match candidate. Second, the two highest-ranked competing EI pairs, which contained one of the two original EIs, had to show a correlation at least 0.05 inferior to the correlation of the original pair.

The statistical significance for the observed preferred direction response in Grik4-positive DS cells (Fig. 4d) has been tested with Student statistic against the null hypothesis of no preferred direction. If Grik4-positive DS cells were expressed homogeneously in the four directions, the differences in cell counts should be accounted for by the statistical fluctuations arising from the optogenetic stimulation efficiency. In order to account for the optogenetic efficiency scaling, we compared the percentage of DS cells responding in each of the four cardinal directions over all DS RGCs. If the null hypothesis were true, the average percentage difference between visually stimulated and optogenetically stimulated DS cells should be compatible with 0.

Differences for each of the 4 cardinal directions were calculated as

Δ = |*D*_*V*_ − *D*_*O*_|, where D_v_ is the visually elicited DS RGCs, in any given direction, as a percentage of all visually elicited DS RGCs. D_O_ is then the percentage of optogenetically elicited DS RGCs over all optogenetically elicited DS RGCs.

A Student t–test was then calculated as follows:

3 $$t=\frac{\bar{{\rm{\Delta }}}-0}{\sigma /\surd n}\cong 2.61\,with\,dof=4,$$

where $\bar{{\rm{\Delta }}}$ is the average difference over all directions, σ is the estimated standard deviation and n = 4 is the number of samples. The null hypothesis can therefore be rejected with a significance level of 0.05.

### Immunohistochemistry

After recording, retinas were carefully removed from the electrode array and fixed for 30 minutes in 4% PFA and washed in PBS with 0.5% Triton X. Retinas were then incubated for 1 day at 4 °C in a blocking solution of PBS and 3% donkey serum, then incubated for 2 days at 4 °C in block solution with primary antibody against tdTomato (Abcam, ab62341) at 1:1000 dilutions. Retinas were then washed in PBS and incubated overnight at 4 °C in fluorescent conjugate secondary antibodies (Life Technologies).

Finally, the tissue was washed and mounted on glass slides with Vectashield mounting medium.

### Imaging and alignment

Epiluorescent images of the retina mounted on the MEA were taken with an Olympus IX71 inverted research microscope (10x magnification). Images were taken after visual stimulation to avoid photoreceptor bleaching that would undermine visual responses, and before optogenetic stimulation to minimize tdTomato bleaching by the intense microLED light. After immunostaining, confocal images of the retina were taken on the Leica SP5 Confocal microscope (60X magnification, z-stack spacing 0.34 μm). Images were stitched together in FIJI using the plugins for either pairwise or grid collection stitching^35^. MEA epifluorescent images and immunostained confocal images were then manually registered by matching the unique pattern of RGC somas’ locations. Matching was done by adjusting position, rotation and scale of one of the images.

### Data availability statement

The datasets generated during the current study are available from the corresponding author on reasonable request.

## Electronic supplementary material

Supplementary Information
