# Supplementary material for: Large scale matching of function to the genetic identity of retinal ganglion cells
Source: Sci Rep. 2017 Nov 13;7:15395. doi: 10.1038/s41598-017-15741-7 (PMC5684394; doi:10.1038/s41598-017-15741-7)
Supplement: Supplementary file 1 — Supplementary Information [file 41598_2017_15741_MOESM1_ESM.pdf]

Large scale matching of function to the genetic identity of retinal ganglion cells

Filippo Pisano*[1], Erin Zampaglione*[2], Niall McAlinden[1], Jennifer Roebber[2], Martin D.Dawson[1], Keith Mathieson[†1], Alexander Sher[†3]

1 *Institute of Photonics, Dept. of Physics, University of Strathclyde, G1 1RD, Glasgow, UK.*

2 Molecular, Cell and Developmental Biology, University of California Santa Cruz, Santa Cruz, CA,US

3 Santa Cruz Institute for Particle Physics, University of California Santa Cruz, Santa Cruz, CA, US

* These authors contributed equally to this work

†These authors jointly supervised the work and are the corresponding authors

Supplementary Figures

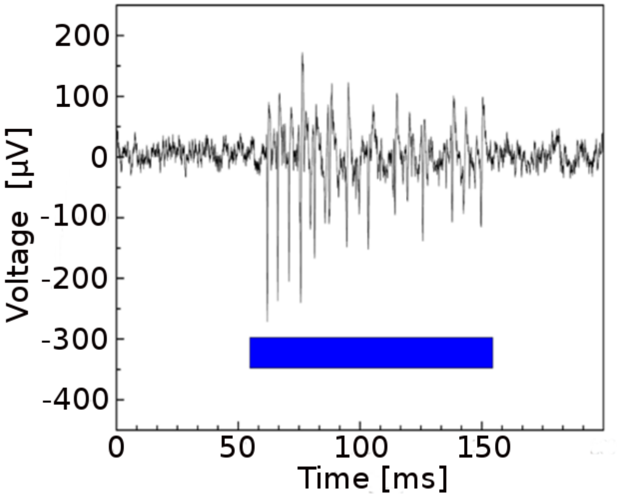

Supplementary figure 1
Example of a raw electrode trace showing an optogenetically induced spike burst. No artefact was observed in connection with the onset/offset of blue light, represented by the blue bar.

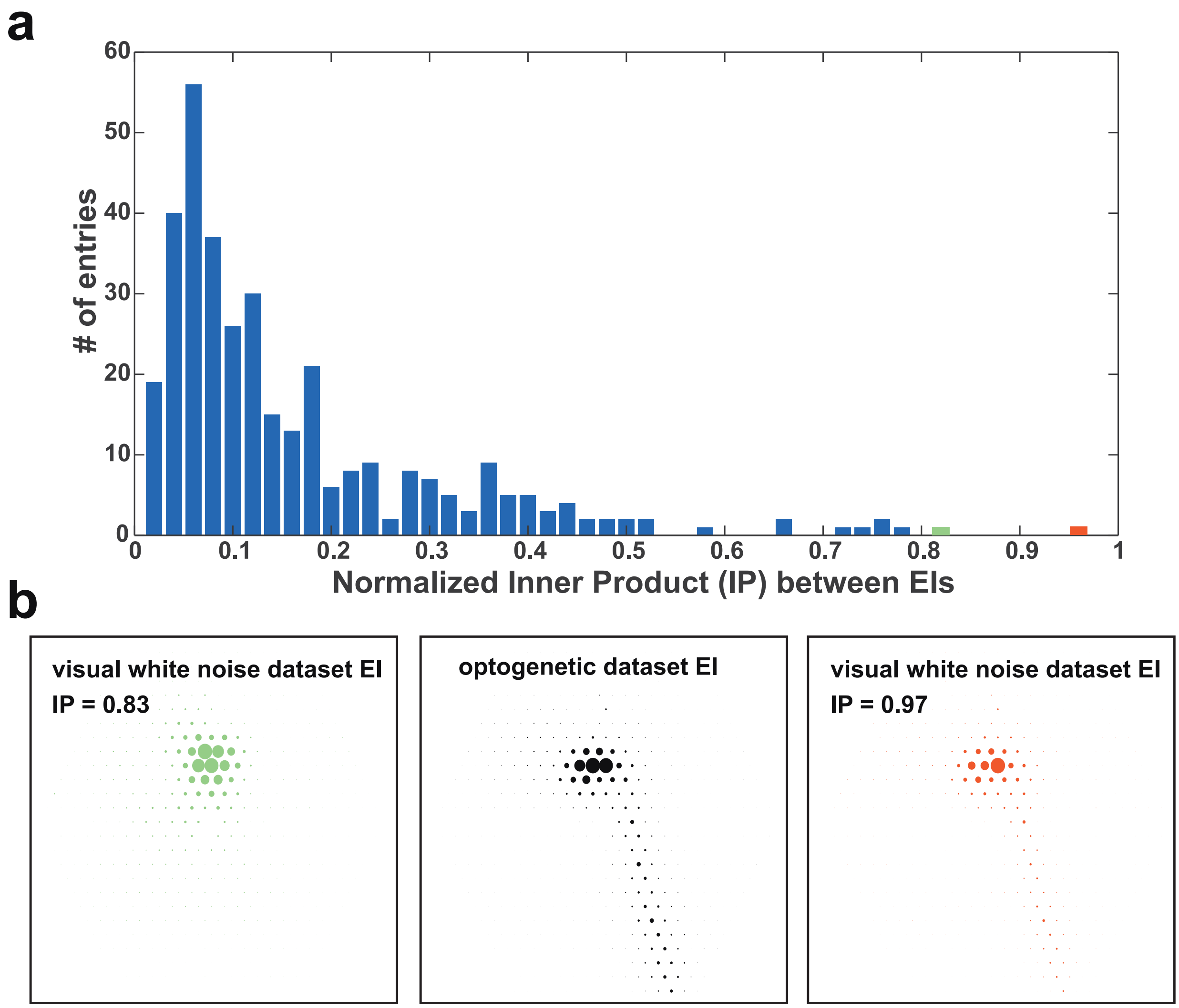

Supplementary figure 2
Example of the EI match between the optogenetically and visually stimulated RGCs. (**a**) Distribution of normalized inner products (see Methods) between the EI of an optogenetically stimulated RGC and EIs of the RGCs in the visually stimulated dataset. The entries colored red and green correspond to the best and the next best matches. (**b**) EIs of the optogenetically stimulated RGC (middle panel), EI of the best matched RGC from the visual dataset (right panel) and EI of the next best matched RGC from the visual dataset (left panel).
